# Phosphorus Nutrition Affects Temperature Response of Soybean Growth and Canopy Photosynthesis

**DOI:** 10.3389/fpls.2018.01116

**Published:** 2018-08-06

**Authors:** Shardendu K. Singh, Vangimalla R. Reddy, David H. Fleisher, Dennis J. Timlin

**Affiliations:** ^1^Adaptive Cropping Systems Laboratory, United States Department of Agriculture, Agricultural Research Service, Beltsville, MD, United States; ^2^School of Environmental and Forest Science, University of Washington, Seattle, WA, United States

**Keywords:** carbon balance, carbon gain, *Glycine max*, phosphorus utilization efficiency, T × P interaction, tissue phosphorus

## Abstract

In nature, crops such as soybean are concurrently exposed to temperature (T) stress and phosphorus (P) deficiency. However, there is a lack of reports regarding soybean response to T × P interaction. To fill in this knowledge-gap, soybean was grown at four daily mean T of 22, 26, 30, and 34°C (moderately low, optimum, moderately high, and high temperature, respectively) each under sufficient (0.5 mM) and deficient (0.08 mM) P nutrition for the entire season. Phosphorus deficiency exacerbated the low temperature stress, with further restrictions on growth and net photosynthesis. For P deficient soybean at above optimum temperature (OT) regimes, growth, and photosynthesis was maintained at levels close to those of P sufficient plants, despite a lower tissue P concentration. P deficiency consistently decreased plant tissue P concentration ≈55% across temperatures while increasing intrinsic P utilization efficiency of canopy photosynthesis up to 147%, indicating a better utilization of tissue P. Warmer than OTs delayed the time to anthesis by 8–14 days and pod development similarly across P levels. However, biomass partitioning to pods was greater under P deficiency. There were significant T × P interactions for traits such as plant growth rates, total leaf area, biomass partitioning, and dry matter production, which resulted a distinct T response of soybean growth between sufficient and deficient P nutrition. Under sufficient P level, both lower and higher than optimum T tended to decrease total dry matter production and canopy photosynthesis. However, under P-deficient condition, this decrease was primarily observed at the low T. Thus, warmer than optimum T of this study appeared to compensate for decreases in soybean canopy photosynthesis and dry matter accumulation resulting from P deficiency. However, warmer than OT appeared to adversely affect reproductive structures, such as pod development, across P fertilization. This occurred despite adaptations, especially the increased P utilization efficiency and biomass partitioning to pods, shown by soybean under P deficiency.

## Introduction

Temperature and nutrient availability are among the main drivers for plant productivity and changes in these factors strongly influence plant adaptation to a given environmental niche. Phosphorus (P) deficiency limits crop productivity in a large area of agricultural lands, and its rapid depletion as a natural resource is a global concern (Vance et al., 2003; Cordell et al., 2009). In addition, agricultural productivity is subjected to environmental warming due to the projected increase in global surface mean air temperature of more than 1.5°C by end of the 21st century (IPCC, 2013). For instance, every 1°C rise in growing season temperature can lead to about a 17% decrease in soybean [*Glycine max* (L.) Merr.] yields in the United States (Lobell and Asner, 2003). Crop yield is vulnerable to the increased seasonal temperature that is often coupled with occurrences of extreme events, such as heat waves (Siebers et al., 2015; Zhao et al., 2017). In nature, plants are simultaneously exposed to multiple environmental drivers and crop productivity can be limited by more than one resource, such as sub- or supra-optimal temperature and inadequate P nutrition (Sinclair, 1992). Normally, evaluation of climate change factors has been done under adequate nutrient supply while a large proportion of crop production might globally occur in nutrient-limited conditions (Sinclair, 1992; Lenka and Lal, 2012). Since temperature is one of the main drivers of crop growth processes, it is unclear how soybean response to temperature will be influenced under P limited conditions. Therefore, T × P interaction studies will help to demonstrate whether the relative response of soybean grown under a range of expected temperature gradients is altered between sufficient and deficient P nutrition.

Soybean is a vital source of protein and vegetable oil, and one of the most widely grown crop worldwide (Conner et al., 2004). The optimum mean temperature range for soybean vegetative and reproductive growth varies between approximately 25–37°C and 22–26°C, respectively (Brown, 1960; Egli and Wardlaw, 1980; Thomas et al., 2010; Hatfield et al., 2011). Temperatures below or above the optimum growth temperature often result in decreased crop productivity due to reduced carbon assimilation and biomass accumulation (Siebers et al., 2015; Xu et al., 2016). P nutrition also has strong impacts on photosynthesis, biomass accumulation and partitioning, and yield and quality (Israel and Rufty, 1988; Jaidee et al., 2013; Singh et al., 2014). Previous studies have shown an approximate decrease of 20–45% in soybean photosynthesis and dry matter, and about a 45–50% reduction in plant tissue P concentration, due to P deficiency when grown under an OT regime (Cure et al., 1988; Singh et al., 2014). However, it is unclear whether sub-optimal plant P concentration will suppress soybean performance similarly when grown under non-optimal temperature regimes. In an interaction study with different CO_2_ levels, non-optimal temperatures and P deficiency independently influenced the soybean response to CO_2_ by decreasing the magnitude of elevated CO_2_-mediated growth stimulation (Singh et al., 2014; Xu et al., 2016). Both elevated CO_2_ and P deficiency have been shown to increase plant tissue P utilization efficiency under optimal temperature regime (Cure et al., 1988; Singh et al., 2014). However, we are not aware of previous studies that evaluated whether additional P fertilization would compensate for high or low T stress or result in improved P utilization efficiencies. Neverthless, application of P fertilizer has been reported to alleviate yield losses, at least partly, in water-stressed soybean (Jin et al., 2006), and a warmer than OT decreased wheat yield regardless of the nitrogen fertilization (Mitchell et al., 1993). Thus, the influence of sub- and supra-optimal temperature on growth and P utilization efficiency of soybean across P nutrition needs yet to be investigated.

Alterations in plant growth and nutrient use response are expected due to interaction with environmental stresses (Sinclair, 1992). Usually, in-season nutrient applications to standing crops are not recommended under adverse climatic conditions (e.g., heat and drought). One of the plausible reasons for this practice is the decreased nutrient demand due to poor crop growth under stress. However, experimental observations have indicated increased uptake and accumulation of nutrient such as P despite the decreased growth in wheat due to HT (Manoj et al., 2012) or in soybean due to water stress (Jin et al., 2006). High temperature has also been shown to increase tissue P concentration of wheat (Manoj et al., 2012). Batten et al. (1986) found increased P uptake and tissue P concentration in wheat when grown in a P deficient medium at HT regimes. Ercoli et al. (1996) reported that HT-mediated increase in sorghum P content was attributable to greater P uptake. Alternatively, P uptake did not increase in *Pinus ponderosa* L. at high air temperature (Delucia et al., 1997). The onset of soybean reproductive growth is often delayed under warmer temperatures (Thomas et al., 2010), while such effects have been not observed under P deficiency (Qiu and Israel, 1994; Cure et al., 1988; Singh et al., 2014). P is an integral part of cellular membranes and nucleic acid, and directly involved in carbohydrate metabolism and protein synthesis, and thus a close relationship is often found between plant tissue P status and various agronomic and physiological traits (Singh and Reddy, 2014, 2015; Singh et al., 2015).

Most of the previous investigations on crops including soybean under different levels of temperature have often been made with adequate nutrient supply (Baker et al., 1989; Campbell et al., 1990; Thomas et al., 2010; Siebers et al., 2015; Xu et al., 2016). However, cold or heat stress can well coincide with crops grown under P-deficient soils. Since, soybean growth is highly sensitive to both P nutrition and temperature that strongly affects plant’s net carbon assimilation and biomass production. Interaction studies will help to elucidate whether combined effects of these factors will exacerbate or alleviate the potential negative impacts on plants. Studies evaluating T × P interaction effects on row crops are extremely limited (Ercoli et al., 1996; Manoj et al., 2012; Suriyagoda et al., 2012) and in soybean not available to the best of our knowledge. Since P fertilization is economically important for farmers and poses risks of environmental pollution, an assessment of temperature response of soybean to varying P nutrition is necessary to predict its adaptation under a changing climate. The objective of this study was to quantify the interactive effects of temperature and P nutrition on soybean dry matter accumulation, biomass partitioning, canopy photosynthesis, and tissue P allocation and utilization.

## Materials and Methods

### Soil-Plant-Atmosphere-Research (SPAR) Facility

The experiment was conducted in sunlit Soil-Plant-Atmosphere-Research (SPAR) chambers located at the USDA-ARS Henry A. Wallace Agricultural Research Center facility in Beltsville, MD, United States. Each SPAR chamber consists of a steel soilbin (1 m deep by 2 m long by 0.5 m wide) to accommodate the root system, and Plexiglas chamber (2.5 m tall by 2.2 m long by 1.4 m wide) to accommodate aerial plant parts, a heating and cooling system, and an environmental monitoring and control system. Plexiglas transmits >90% of the ambient solar radiation inside SPAR (Kim et al., 2007). Chambers are sealed as tightly as possible to minimize gas exchange with outside air. The details of the SPAR chambers and methods of operation and monitoring have been described previously (Fleisher et al., 2009; Timlin et al., 2017).

Air temperature is controlled by cooling and heating of air inside the chambers. Chilled ethylene glycol is supplied to the cooling system via electronic valves depending on cooling requirements. Electrical resistance heaters provide pulses of heat, as needed, to fine-tune the air temperature and help control vapor pressure deficit (VPD). The air passes over cooling coils and heating elements through the top portion inside chambers with a sufficient velocity to cause leaf flutter (2.5 m s^-1^) and returns to the air-handling unit just above the soil level. The condensate from cooling coils of each SPAR is automatically collected and weighed every 15 min interval via a pressure transducer and solenoid valves to estimate the canopy evapotranspiration (ET) (Baker et al., 2004; Timlin et al., 2007). Each chamber is equipped with an infrared gas analyzer (LI-6262, LI-COR Inc., Lincoln, NE, United States) and a gas mass flow controller (FM-766, Omega Engineering, Stamford, CT, United States) to measure and control CO_2_ injection. Carbon dioxide leakage rates are measured daily by injecting nitrous oxide (N_2_O) and measuring the decay (Baker et al., 2004). Automated, continuous monitoring (10 s interval) and measurement (5 min intervals logging) of all-important environmental variables including temperature, CO_2_ and irrigation, and plant canopy gas exchange variables in each chamber was controlled by a dedicated microcomputer workstation using a custom program (Baker et al., 2004; Fleisher et al., 2009).

### Plant Culture and Treatments

Soybean [*Glycine max* (L.) Merr., cv. NC-Roy] seeds were sown in soil bins filled with a manufactured soils consisting of 75% quarry sand and 25% vermiculite by volume in eight SPAR chambers on 17 June 2015. Soybean was planted in nine 50-cm rows (20 cm apart) with five plants per row. Seedling emergence was observed 6 DAP. Two plants from the middle of each row (total 18 plants) were harvested at 29 DAP, and four rows each with three plants (total 12 plants) were harvested at 48 DAP to avoid plant competition and to determine aboveground dry mater components at early growth periods. Thus, five rows (40 cm apart) with three plants each (total 15 plants m^-2^) were retained in each SPAR after 48 DAP. Two rows (total six plants) from the middle of each chamber was again harvested at 90 DAP leaving nine plants m^-2^ until the final harvest, which was completed at 118 DAP. Row spacing, plant densities, and row and/or plant harvests during each harvest period were designed so as to maintain a uniform space around each plant.

After emergence, treatments were initiated in the combination of 4 day/night temperatures (T) as 24/18°C (moderately low, MLT), 28/22°C (optimum, OT), 32/26°C (moderately high, MHT), 36/30°C (HT) each under two levels of P nutrition as 0.5 mM (sufficient) and 0.08 mM (moderately P-deficient) supplied in a modified Hoagland’s nutrient solution as fertigation (Hewitt, 1952). The target P nutrition levels were obtained using ammonium phosphate (NH_4_H_2_PO_4_) and additional triple superphosphate [TSP, (Ca(H_2_PO_4_)2H_2_O)] to maintain same N levels in both solutions. The fertigation solution was applied 4–6 times per day in excess of the evapotranspiration rate using separate solution tanks for both P treatments. The excess fertigation drained from the soil through outlets at the bottom of each soil bin. The daytime temperature was controlled in square-wave fashion and initiated at sunrise and returned to the nighttime temperature 1 h after sunset resulting in to 16/8 h day/night T. Temperature was controlled at the treatment’s set point and exhibited the seasonal average daily mean temperature 22 ± 0.13, 26.1 ± 0.12, 29.9 ± 0.14, and 33.8 ± 0.12°C for the MLT, OT, MHT, and HT treatments, respectively, averaged across P nutrition. The seasonal mean CO_2_ was 419 ± 13 μmol mol^-1^ averaged across treatments during daylight hours. Relative humidity in the SPAR chambers varied between 46 and 68% during the experiment. A list of the measured controlled environment variables and VPD for each treatment is provided in **Table 1**. The VPD was determined following Murray (1967).

**Table 1:** Phosphorus (P) nutrition levels, day/night air temperature (T), daily mean T, and CO_2_ concentration, and the measured daily mean T, vapor pressure deficit (VPD), and CO_2_ concentration inside the chambers.

Treatments				Measured variables		
P (mM)	T, day/night (°C)	Daily mean T (°C)	CO_2_ (μmol mol^-1^)	Daily mean T (°C)	VPD (kPa)	CO_2_ (μmol mol^-1^)
0.08	24/18	22	400	22.0 ± 0.15	1.10 ± 0.08	418 ± 11
	28/22	26	400	26.2 ± 0.11	1.34 ± 0.12	415 ± 11
	32/26	30	400	30.0 ± 0.15	2.36 ± 0.24	418 ± 13
	36/30	34	400	33.8 ± 0.12	2.47 ± 0.23	423 ± 12
0.50	24/18	22	400	22.1 ± 0.11	1.15 ± 0.07	417 ± 11
	28/22	26	400	26.1 ± 0.13	1.40 ± 0.12	422 ± 14
	32/26	30	400	29.8 ± 0.14	1.32 ± 0.08	422 ± 12
	36/30	34	400	33.8 ± 0.12	2.53 ± 0.31	420 ± 19

*Each measured value represents seasonal mean ± standard deviation (*n* = 109) between emergence to the end of experiment*.

### Plant Growth Measurements

Plant heights (PH), mainstem node numbers (MSNN) and leaf length at each node were determined on the same seven plants 13 times between 12 DAP and 63 DAP in each treatment. Thereafter, measurement of leaf length was stopped to avoid plant canopy disturbance, but an additional three measurements of PH and MSNN were made before the end of experiment. The leaf length measurements were converted to mainstem leaf areas (MSLA) by developing a relationship between lengths of different leaves and leaf area measured using a LI-3100 leaf area meter (LICOR, Inc., Lincoln, NE, United States). The leaves were obtained from plants during separate destructive harvests for each temperature treatment. Leaf area was calculated using the polynomial second order equation, *y* = 1.0222*x*^2^–0.0825 (*r*^2^ = 0.94, *P* = < 0.0001; *n* = 114; 24/18°C), *y* = 1.0372*x*^2^–0.515 (*r*^2^ = 0.91, *P* = < 0.0001; *n* = 305; 28/22°C), *y* = 0.8569*x*^2^ + 0.9318 (*r*^2^ = 0.90, *P* = < 0.0001; *n* = 326; 32/26°C), and *y* = 0.8885*x*^2^ + 1.1485 (*r*^2^ = 0.93, *P* = < 0.0001; *n* = 165; 36/30°C) where *Y* is area in cm^2^ and *x* is the length in cm. At all four destructive harvests, each plant was separated into the components (e.g., leaves, stems, pods) and leaf area and dry weights of each component were determined by placing samples in air-forced ovens maintained at 70°C until constant weight was achieved. The total dry weight (TDwt) represented the sum of the dry weights of all plant components. The soybean reproductive developmental stages were determined according to Fehr et al. (1971).

### Determination of Tissue Phosphors Concentration

The dry plant components (e.g., leaves, stems, pods) were ground using a Wiley Mill (Wiley^®^ Mill, Thomas Scientific, Swedesboro, NJ, United States) to pass through a 1 mm screen. The tissue P concentration (mg P g^-1^ dry weight) of each plant component was determined at the Agriculture Diagnostic Laboratory, University of Arkansas, Fayetteville, AR, United States, using a standard procedure (Plank, 1992). The weighted whole-plant P concentration was estimated as the sum of the products of the dry mass of plant components and their P concentration divided by total dry weight (Singh et al., 2015).

### Canopy Gas Exchange Measurements

The CO_2_ exchange rate (CER) during daytime represents canopy net photosynthesis for the entire plant population in a chamber (*P*_net_, μmol CO_2_ m^-2^ s^-1^) and was derived based on the amount of CO_2_ injected into the chamber and the CO_2_ not used by plants or lost due to leakage (Baker et al., 2004; Timlin et al., 2017). The canopy gross photosynthesis (*P*_G_) was estimated as *R*_D_ + *P*_net_ where *R*_D_ (μmol CO_2_ m^-2^ s^-1^) is the total respiration. *R*_D_ was calculated from CER values for both daytime and nighttime temperatures. Daytime *R*_D_ was obtained between 21:00 h and 22:00 h each day when solar radiation was negligible, and CER values obtained between 1:00 to 5:00 h were used for *R*_D_ for nighttime temperatures as described by Fleisher et al. (2008). Although the *R*_D_ does not account for photorespiration, this method has successfully been used to relate canopy photosynthesis with dry matter production (Reddy et al., 1991; Dutton et al., 1988; van Iersel and Kang, 2002; Fleisher et al., 2008; Timlin et al., 2017). The canopy CER data were averaged at 15 min intervals and modeled using the non-linear rectangular hyperbola function (Loomis and Connor, 1996).

(1) $$P_{G}\;=\;\frac{a\;\;\times \;PAR\;\;\times \;P_{max}}{\left(a\;\;\times \;PAR\right)\;+\;P_{max}}$$

where *P*_max_ is the asymptotic rate of *P*_G_, PAR is the PAR (μmol m^-2^ s^-1^), and *a* is a coefficient (μmol CO_2_ m^-2^ s^-1^). The PROC NLIN procedure of SAS (SAS Enterprise Guide, 4.2, SAS Institute Inc., Cary, NC, United States) was used to obtain these parameters by Gauss-Newton iteration method using equation [1]. This procedure was necessary to smooth CER data, interpolate missing data when SPAR chamber doors were open to take measurements, and to calculate *P*_G_ at a given PAR level (e.g, *P*_G_1500 at 1500 μmol m^-2^ s^-1^ PAR) (Timlin et al., 2017). The weekly or total net C-gain (g C m^-2^) was calculated as the sum of the weekly or seasonal integral *P*_net_ (in mol CO_2_ m^-2^) multiplied by 12.0107 (g). The value 12.0107 represents the mass of carbon in one mole of CO_2_.

### Data Analysis

**FIGURE 1:**
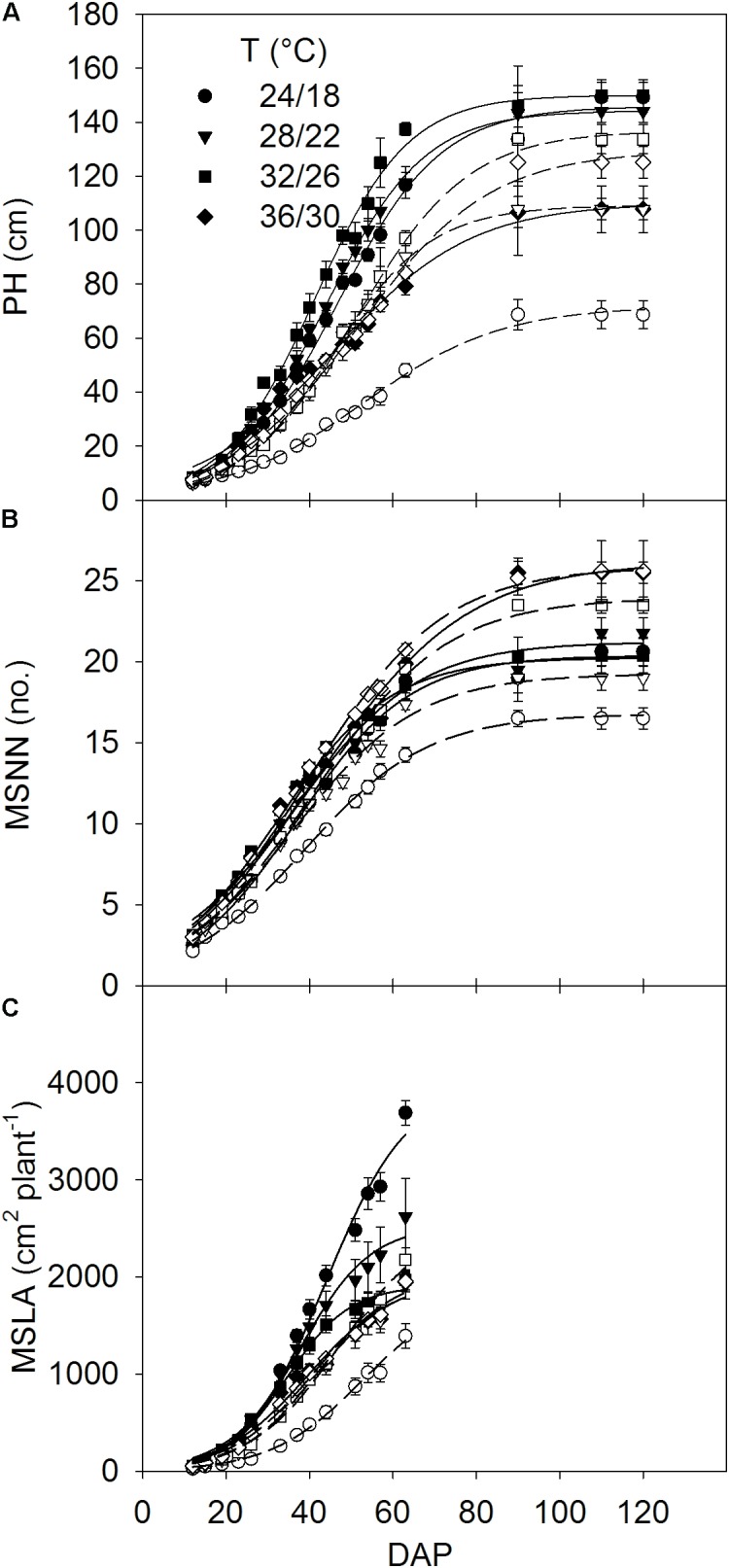
Seasonal pattern of **(A)** plant height (PH) and mainstem **(B)** node numbers (MSNN) and **(C)** leaf area (MSLA) across DAP of soybean grown at four temperatures (T, day/night) under phosphorus (P) sufficient (0.50 mM P, filled symbols) and P-deficient (0.08 mM P, unfilled symbols) conditions. Symbols represent the mean ± se of seven plants. Smooth lines through the data points are presented. Error bars smaller than the symbol are not visible.

Relationships of PH, MSNN, and MSLA with DAP were used to estimate the rates of MSER, MNAR and MLAER, respectively, as described by Singh et al. (2017). Since rapid change of these traits was mainly associated with the linear part of curves residing between 19 and 54 DAP (**Figure 1**), the average rate in this period was taken as a representative of the maximum MSER, MNAR, and MLAER. The intrinsic P utilization efficiency (IPUE) for the seasonal total *P*_net_ was calculated by dividing the total seasonal P_net_ (mol CO_2_ m^-2^) with the averaged plant P concentration (mg g^-1^) (Singh et al., 2015).

Statistical analyses were performed using SAS (SAS Enterprise Guide, 4.2, SAS Institute Inc., Cary, NC, United States). A prior uniformity study using the same chambers (Fleisher et al., 2009) indicated that no statistical differences were present among SPAR chambers. Therefore, to test for the effect of treatments and their interaction on growth measurements, PROC MIXED with Kenward-Rogers (kr) adjustment of degrees of freedom was used for analysis of variance using the individual plant as pseudoreplicates. The treatment comparisons were conducted by least square means (LSMEANS) procedure (at α = 0.05) when the interaction was significant at *P* ≤ 0.05 with the letter grouping obtained using pdmix800 macro (Saxton, 1998), and the averaged least significant difference (LSD) is also presented. When the interaction was not significant, mean values across a given treatment (P or T) are given. The regression analysis was conducted using PROC REG procedure of SAS and SigmaPlot (Version 11.0 Systat Software Inc., San Jose, CA, United States).

## Results

### Soybean Reproductive Development

Flower initiation (R1 stage) started about 56–61 DAP at OT across both P levels (**Table 2**). Relative to OT, the colder and two warmer temperatures delayed the R1 stage by 3–5 and 8–14 days, respectively, with the greatest delay observed at HT under P-deficient condition. Soybean grown under MLT and OT reached R2 stage at same period, which differed between P-sufficient and deficient conditions (64 and 69 DAP, respectively). Across P levels, the periods between R1 and R2 stages were 3–8 days for MLT and OT, 5–11 days for MHT, and 10–20 days for HT. However, plants grown at the combination of HT and P deficiency stayed between R1 and R2 stage for the longest period (20 days). There was no pod formation at the two warmer temperatures at 90 DAP (**Table 2**). However, all treatments had pods and seeds at the final harvest (118 DAP).

**Table 2:** Effect of temperature (T, day/night, °C) and phosphorus (P, mM) treatments on the reproductive development of soybean growth.

Treatments		Days from planting to			Time Period	
P	T	R1	R2	R3	R1–R2	R2–R3
0.08	24/18	61	64	<90	3	<26
	28/22	56	64	<90	8	<26
	32/26	64	69	>90	5	–
	36/30	70	90	>90	20	–
0.50	24/18	64	69	<90	5	<21
	28/22	61	69	<90	8	<21
	32/26	64	75	>90	11	–
	36/30	69	79	>90	10	–

*These observations were made 3–4 times in a week on all plants in each treatment just before flowering to 80 DAP and on the destructive harvest at 90 DAP. The developmental stages were determined based on Fehr et al. (1971)*.

### Tissue Phosphorus Concentrations

The effect of temperature on tissue P concentration was mainly significant for plant components at the final harvest (118 DAP) (**Table 3**). At this stage, P deficient plants at HT consistently had a higher tissue P concentration in stems, leaves, and pods compared with plants at optimal temperature (**Table 3**). However, this temperature response was not consistent under sufficient P nutrition. A distinct temperature response between P treatments led to the observed significant T × P interactions for the tissue P concentration in plant components, especially, at 118 DAP (**Table 3**). Regardless of harvest dates, P deficiency significantly decreased plant P concentration ≈55% averaged across temperature treatments as compared with sufficient P level. The average decline of P concentration under P deficiency was greater in stems (68.8%) than leaves (50.1%) followed by pods across temperatures and harvest dates. Leaves also tended to have greater P concentration than stems under P-deficient conditions. Among plant components, pods showed the greatest P concentration (**Table 3**).

**Table 3:** Effect of temperature (T, day/night, °C) and phosphorus (P, mM) treatments on the tissue P concentrations (mg g^-1^ dry weight) of plants, leaves, stems, and pods at 29, 90, and 118 days after planting (DAP).

Treatment		29 DAP			90 DAP			118 DAP			
P	T	Plant	Leaves	Stems	Plant	Leaves	Stems	Plant	Leaves	Stems	Pods
0.08	24/18	1.88	1.91	1.81	2.83	2.78	1.72	2.82	1.22d	0.55d	4.83cd
	28/22	1.81	1.96	1.54	2.75	3.87	2.04	2.21	1.68d	0.92d	3.20e
	32/26	1.76	1.91	1.37	3.04	3.58	2.72	2.65	2.49c	1.96c	4.25d
	36/30	1.94	2.14	1.51	3.47	4.43	2.95	3.02	3.12bc	2.77c	4.40d
0.50	24/18	4.74	4.79	4.64	6.31	6.01	6.29	5.56	4.21a	5.01b	6.30a
	28/22	4.95	5.30	4.40	6.80	5.78	7.27	5.89	4.25a	7.12a	5.38bc
	32/26	4.81	5.22	4.26	6.66	5.97	6.97	6.94	3.80ab	7.73a	5.93ab
	36/30	5.03	5.30	4.52	6.18	6.53	5.95	6.13	4.45a	7.22a	4.89cd
	LSD	0.46	0.52	0.49	0.97	0.91	1.72	0.93	0.81	0.82	0.68
ANOVA^†^	T	0.5954	0.2220	0.1424	0.8288	0.0191	0.5135	0.0931	0.0085	<0.0001	0.0004
	P	<0.0001	<0.0001	<0.0001	<0.0001	<0.0001	<0.0001	<0.0001	<0.0001	<0.0001	<0.0001
	T × P	0.8209	0.5627	0.9524	0.2593	0.179	0.3021	0.1126	0.0139	0.0139	0.0124
**Mean values across each treatment^††^**								
P treatment	0.08	1.85*	1.98*	1.56*	3.02*	3.67*	2.36*	2.68*	–	–	–
	0.50	4.88	5.15	4.46	6.49	6.07	6.62	6.13	–	–	–
T treatment	24/18	3.31	3.35	3.23A	4.57	4.40B	4.01	4.19AB	–	–	–
	28/22	3.38	3.63	2.97AB	4.78	4.83B	4.66	4.05B	–	–	–
	32/26	3.29	3.57	2.82B	4.85	4.78B	4.85	4.80A	–	–	–
	36/30	3.49	3.72	3.02A	4.83	5.48A	4.45	4.58AB	–	–	–

^†^*The significance test (*P*-values) of the analysis of variance (ANOVA) between T and P are given. When the T × P interaction is significant, within a column means followed by the same lowercase letter are not significantly different. ^††^When the T × P interaction is not significant, means across each treatment is given; the symbol ^∗^ indicate significant difference between the P treatments; the mean value between T treatments followed by same uppercase letter is not significant. Data are the mean (*n* = 3). LSD, least significant difference at α = 0.05*.

### Plant Growth and Dry Matter Production

**FIGURE 2:**
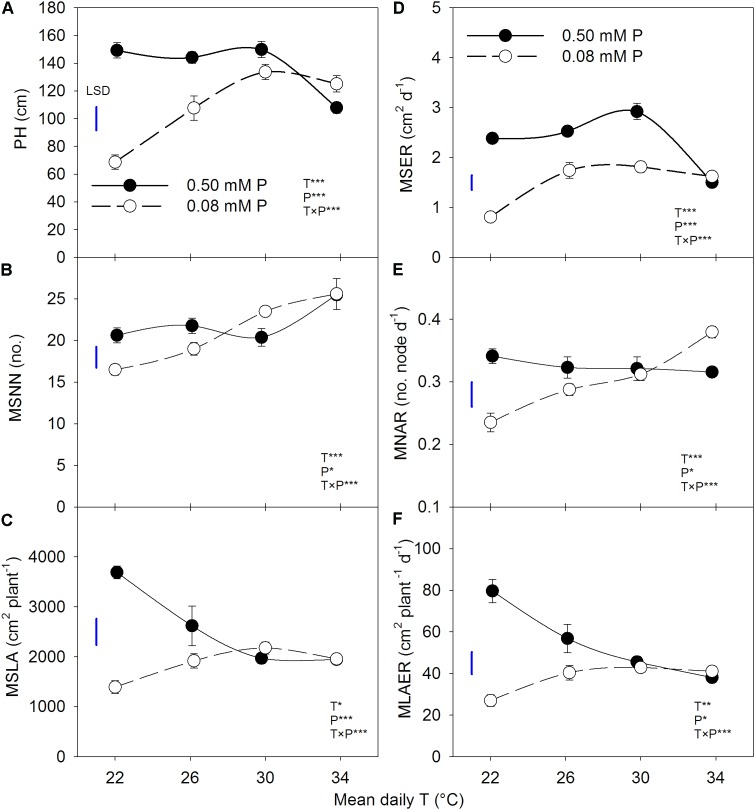
Temperature (T) response of **(A)** plant height (PH) and **(B)** mainstem node number (MSNN) at 118 DAP and **(C)** leaf area (MSLA) at 63 DAP, **(D)** mainstem elongation rate (MSER), **(E)** mainstem node addition rate (MNAR), and mainstem **(F)** leaf area expansion rate (MLAER) of soybean grown at four temperatures under phosphorus (P) sufficient (0.50 mM P, filled symbols) and P-deficient (0.08 mM P, unfilled symbols) conditions. Symbols represent the mean ± se of seven plants. Smooth lines through the data points are presented. The analysis of variance for the effect of treatments and their interaction (T, P, T × P) is also shown by the significance levels where ^∗^, ^∗∗^, and ^∗∗∗^ represent *P* ≤ 0.05, *P* ≤ 0.01, and *P* ≤ 0.001, respectively. The magnitude of the least significant difference (LSD) is shown by the length of the vertical line. Error bars smaller than the symbol are not visible.

The plant height, MSNN, and MSLA followed a sigmoidal growth pattern exhibiting a rapid growth between 19 and 54 DAP across treatments (**Figure 1**). Plant height and MSNN appeared to reach saturation before 90 DAP and did not show substantial increases thereafter. There was significant T × P interaction for the final plant height, MSNN, MSLA, and growth rate parameters (MSER, MNAR, and MLAER, **Figure 2**). Under sufficient P level, HT had the lowest (107 cm) plant height while values in other temperature treatments were similar (≈147 cm) (**Figure 2A**). In contrast, under P-deficient conditions, plant height increased from 68.6 to 133 cm as temperature increased from MLT to MHT then declined to 125 cm at HT. The MSNN also showed a similar pattern but did not decrease at HT under P deficiency (**Figure 2B**). The final plant height, MSNN, and MSLA of P-deficient plants were consistently lower at and below the OT compared with plants under sufficient P level (**Figures 2A–C**). A similar pattern was also observed for growth rates (MSER, MNAR, and MLAER) which exhibited distinctive temperature responses between P levels (**Figures 2D–F**).

There was significant T × P interaction for TLA and TDwt at early growth periods corresponding to 29 and 48 DAP (**Table 4**). However, at the last two sampling dates (90 and 118 DAP), a significant temperature effect or T × P interaction was mainly observed for TLA. At 118 DAP, TLA, and TDwt were lower at and below OT compared with warmer temperatures (MHT and HT).

**Table 4:** Effect of temperature (T, day/night, °C) and phosphorus (P, mM) treatments on the total leaf area (TLA, cm^2^ plant^-1^), and total dry weight (TDwt, g plant^-1^) at 29, 48, 90, and 118 DAP.

Treatment		29 DAP		48 DAP		90 DAP		118 DAP	
P	T	TLA	TDwt	TLA	TDwt	TLA	TDwt	TLA	TDwt
0.08	24/18	140^e^	0.87^e^	759^d^	4.60^c^	7105	71.1	2960^b^	97.2
	28/22	340^d^	1.94^d^	1720^bc^	10.32^b^	9421	74.1	3527^b^	116.6
	32/26	309^d^	1.72^d^	1853^bc^	11.12^b^	14501	98.3	11926^a^	163.1
	36/30	492^c^	3.09^c^	1508^bcd^	12.27^b^	6204	61.2	11169^a^	155.1
0.50	24/18	667^b^	3.33^bc^	2853^a^	14.71^ab^	7269	59.6	7679^ab^	155.9
	28/22	787^a^	4.02^ab^	2228^ab^	11.78^b^	6071	55.8	10684^a^	180.1
	32/26	739^ab^	4.22^a^	3052^a^	19.41^a^	12035	102.0	7091^ab^	130.4
	36/30	643^b^	4.36^a^	1161^cd^	10.71^b^	7048	66.1	9001^ab^	111.1
	LSD	101	0.64	746	4.60	6953	49.9	6592	110.0
ANOVA^†^	T	0.0002	<0.0001	0.0032	0.0212	0.0342	0.1758	0.1947	0.9484
	P	<0.0001	<0.0001	<0.0001	0.0007	0.4874	0.6958	0.5249	0.7176
	T × P	<0.0001	0.0594	0.0007	0.0054	0.7905	0.9097	0.0290	0.4741
**Mean values across each treatment^††^**								
P treatments	0.08	–	1.91^∗^	–	–	9308	76.2	–	133.0
	0.50	–	3.98	–	–	8106	70.9	–	144.4
T treatments	24/18	–	2.10^C^	–	–	7187^B^	65.4	–	126.6
	28/22	–	2.98^B^	–	–	7746^B^	65.0	–	148.4
	32/26	–	2.97^B^	–	–	13268^A^	100.2	–	146.8
	36/30	–	3.73^A^	–	–	6626^B^	63.7	–	133.1

^†^*The significance test (P-values) of the analysis of variance (ANOVA) between T and P are given. When the T × P interaction is significant, within a column means followed by the same lowercase letter are not significantly different. ^††^When the T × P interaction is not significant, means across each treatment is given; the symbol ^∗^ indicate significant difference between the P treatments; the mean value between T treatments followed by same uppercase letter is not significant. Data are the mean (n = 18, 12, 6, and 9 at 29, 48, 90, and 118 DAP, respectively). LSD, least significant difference at α = 0.05*.

### Biomass Partitioning Response

**FIGURE 3:**
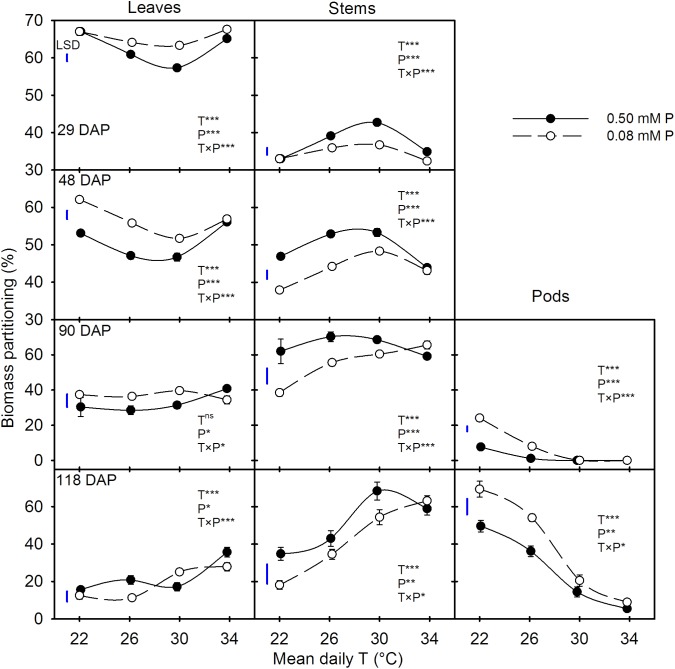
Temperature (T) response of the biomass partitioning to leaves, stems, and pods (%) during destructive harvests made at four different days after planting (DAP) of soybean grown under phosphorus (P) sufficient (0.50 mM P, filled symbols) and P-deficient (0.08 mM P, unfilled symbols) conditions. Symbols represent the mean ± se of 15, 12, 6, and 9 plants at 29, 48, 90, and 118 DAP, respectively. Smooth lines through the data points are presented. The analysis of variance for the effect of treatments and their interaction (T, P, T × P) is also shown by the significance levels, where ^∗^, ^∗∗^, ^∗∗∗^, and ^ns^ represent *P* ≤ 0.05, *P* ≤ 0.01, *P* ≤ 0.001, and *P* > 0.05 (non-significant), respectively. The magnitude of the least significant difference (LSD) is shown by the length of the vertical line. Error bars smaller than the symbol are not visible.

There were significant T × P interactions for biomass partitioning to plant components across all harvest dates (**Figure 3**). Regardless of treatments, the biomass partitioning to leaves and stems decreased as the plants aged. However, partitioning to pods increased between 90 and 118 DAP. At early stages of plant growth (29 and 48 DAP), biomass partitioning to leaves was greater than to stems (average 60% versus 40%), but the opposite was observed for the later two harvest dates. In addition, there were distinct temperature responses for biomass partitioning between leaves and stems, which was more pronounced at earlier harvests (**Figure 3**). Temperature effects on biomass partitioning also differed between P levels. Except at HT (mean daily T of 34°C), the biomass partitioning increased in leaves and pods while decreased in stems under the P-deficient versus sufficient condition (**Figure 3**).

### Canopy Gas Exchange and Total Carbon Gain

**FIGURE 4:**
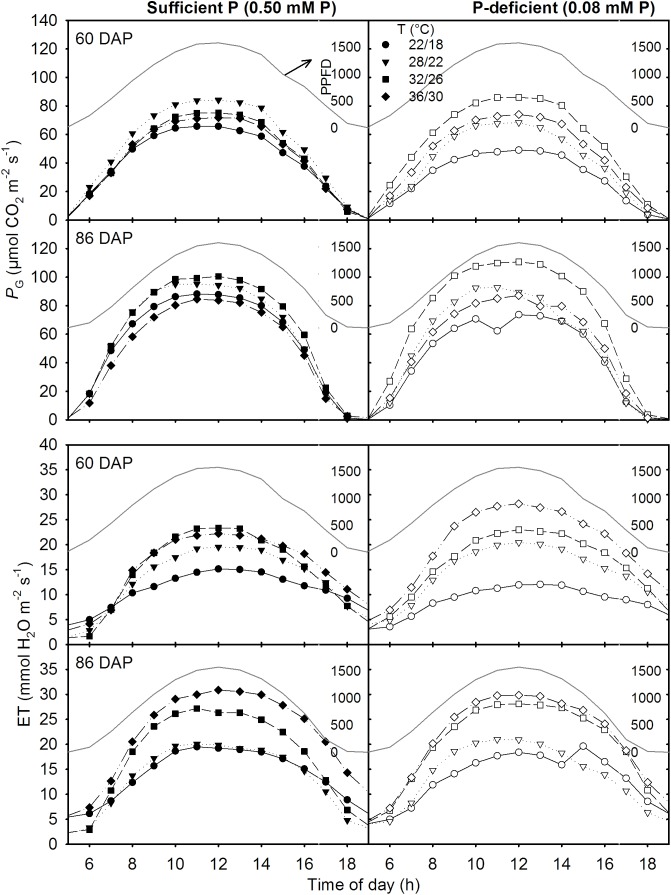
Diurnal canopy gross photosynthesis rate (*P*_G_) and evapotranspiration (ET) for two representative sunny days (60 and 86 days after planning, DAP) of soybean grown at four temperature (T, day/night) treatments under phosphorus (P) sufficient (0.50 mM, left panel) and P-deficient (0.08 mM P, right panel) conditions. Data points are hourly intervals for a given treatment and represents an experimental unit. The PPFD (dark grayed line) is shown for both days as PAR (μmol PAR m^-2^ s^-1^).

**FIGURE 5:**
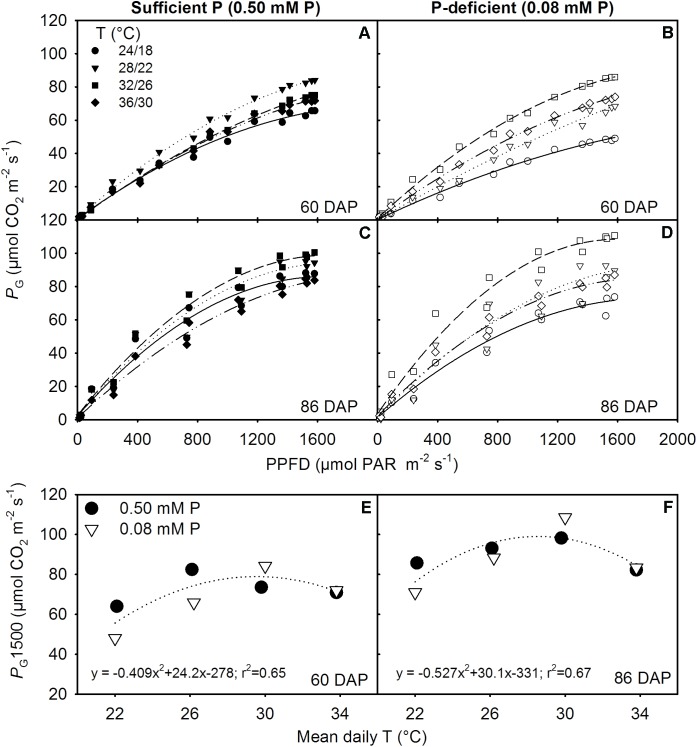
Photosynthetic light response curve (LRC) representing canopy gross photosynthesis rate (*P*_G_) versus photon flux density (PPFD) for two representative sunny days **(A,B)** 60 and **(C,D)** 86 days after planning (DAP) and **(E,F)** the temperature (T) response of *P*_G_ at saturating light (1500 μmol m^-2^ s^-1^ PPFD, *P*_G_1500) for soybean grown at four temperatures (T, day/night) under phosphorus (P) sufficient (0.50 mM P, filled symbols) and P-deficient (0.08 mM P, unfilled symbols) conditions. Data points represent hourly mean for the day (*P*_G_) or the experimental unit (*P*_G_1500) for a given treatment. The regression fit was significant as *P* = 0.072 **(E)** and *P* = 0.060 **(F)**.

**FIGURE 6:**
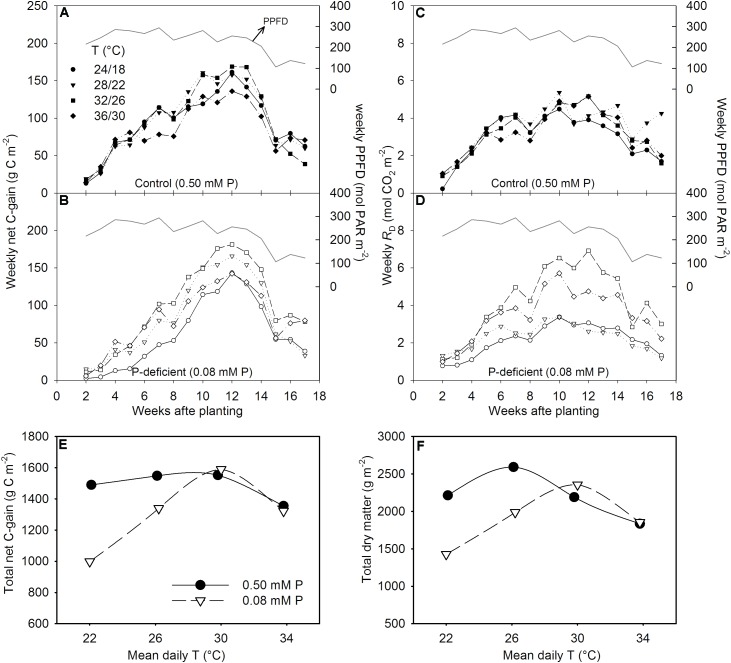
Weekly sum of net C-gain over the season **(A,B)**, total day respiration (*R*_D_) **(C,D)**, and the temperature (T) response of the seasonal total net C-gain **(E)** and total dry matter harvested **(F)** of soybean grown at four temperatures (day/night) under phosphorus (P) sufficient (filled symbols) and P-deficient (unfilled symbols) conditions. The daily PPFD (solid line, **A–D**) over the season is shown as PAR. Data represent an experimental unit for a given treatment.

The canopy *P*_G_ and ET followed a diurnal trend of light intensity (PPFD) exhibiting maximum values around midday (**Figure 4**). ET was often low at MLT but was higher at the two warmer temperatures than OT across P levels. The greatest temperature sensitivity of *P*_G_ was consistently observed at the highest PPFD level across treatments (**Figure 4**). However, under P deficiency, the temperature sensitivity of *P*_G_ appeared to be relatively more pronounced even at low PPFD (e.g., 500 μmol m^-2^ s^-1^) exhibiting a greater variation of *P*_G_ among temperature treatments (**Figures 5A–D**). The *P*_G_ estimated at 1500 μmol m^-2^ s^-1^ PPFD (*P*_G_1500) exhibited a curvilinear increasing response pattern as temperature increased (**Figure 5E,F**). The weekly C-gain (g C m^-2^ week^-1^) over the season also followed a similar pattern as of the PPFD (**Figure 6A,B**). C-gain peaked in the 12th week after planting across treatments. During the same period, MHT had the maximum rate of C-gain across both P levels. At this period, HT and MLT had the lowest rate of C-gain under sufficient and deficient P levels, respectively (**Figure 6A,B**). Moreover, temperature sensitivity for C-gain was greater under P-deficient condition exhibiting relatively larger differences between temperature treatments (**Figure 6B**). The *R*_D_ varied little across temperatures under control P treatment (**Figure 6C**). However, compared to MLT and OT, *R*_D_ was almost doubled at warmer temperatures (i.e., MHT and HT) under P deficiency, especially, between 10 and 12 weeks after planting (**Figure 6D**). The total seasonal net C-gain followed an almost similar pattern as total dry matter harvested for the entire season across treatments (**Figures 6E,F**). Relative to the OT, total net C-gain and dry matter at HT was lower or almost similar under sufficient and deficient P levels, respectively. In respect to P effects, P deficiency had smaller values of total net C-gain (13–33%) and total dry matter (14–26%) at and below OT. However, at warmer than OT both parameters were almost similar to the observations made under sufficient P condition (**Figures 6E,F**).

### Phosphorus Use Efficiency

**FIGURE 7:**
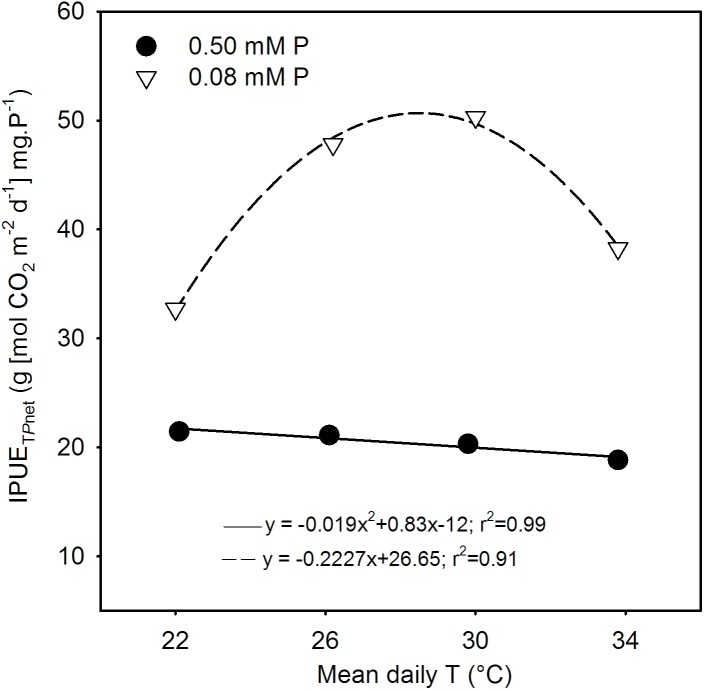
Temperature (T) response of intrinsic phosphorus utilization efficiencies (IPUE) of total net photosynthesis (IPUE_T_*_P_*_net_) of soybean grown under phosphorus (P) sufficient (filled symbols) and P-deficient (unfilled symbols) conditions. Data are the experimental unit for each treatment. The regression fit was significant as *P* = 0.06 (P sufficient) and *P* = 0.007 (P-deficient).

The seasonal intrinsic phosphorus utilization efficiency of total *P_net_* (IPUE_T_*_P_*_net_) was always greater (52–147%) under P deficiency across temperature treatments compared with sufficient P level (**Figure 7**). The temperature response also differed significantly between P nutrition levels. There was a linear decreasing in the control and a in the P-deficient treatment. Under P deficiency, IPUE_T_*_P_*_net_ increased 46–54% consistently between MLT and MHT from an initial value of 32.7 [g (mol CO_2_ m^-2^ s^-1^) mg.P^-1^] then declined about 17% at HT while still exhibiting greater than the initial value.

### Carbon Balance

**FIGURE 8:**
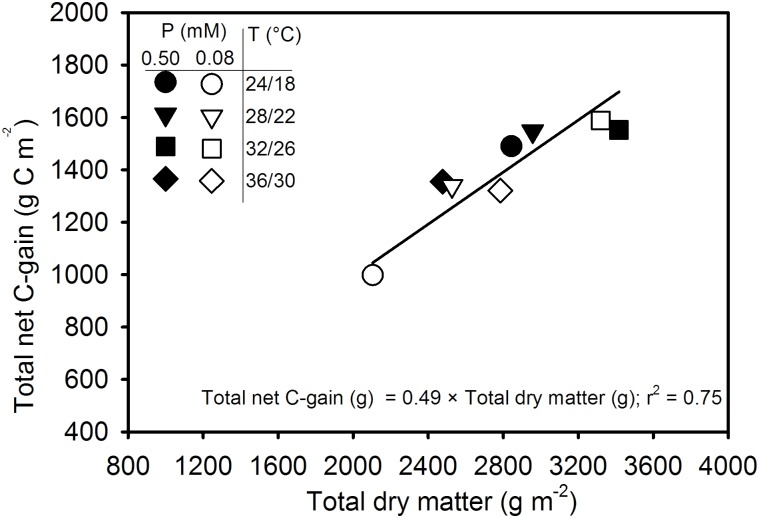
Carbon balance comparing the seasonal total net C-gain from canopy photosynthesis with the total aboveground dry matter produced across temperature (T, day/night) and phosphorus (P) treatments. Data represent an experimental unit for each treatment. The regression fit was significant as *P* ≤ 0.0001.

**Figure 8** reflects the relationship of total net C-gain that can be achieved by canopy photosynthesis with the total dry matter production for an entire season. The total dry matter accounts for aboveground parts of the entire plant population matching with the concurrent *P*_net_ throughout the season. The regression relationship was positive and linear and showed a good agreement (*y* = 0.39*x* + 288; *r*^2^ = 0.80, *P* = 0.0026) when fitted across treatments. The intercept (288) of the linear relationship did not differ significantly (*P* > 0.10) from zero. Therefore, regression relationship was re-fitted after allowing the intercept to pass through zero and a new set of regression coefficients was obtained as *y* = 0.49*x*; *r*^2^ = 75; *P* < 0.0001). The slope of regression relationship 0.49 indicated that the carbon fixed via canopy photosynthesis accounted close to half of the total above-ground dry matter production across the treatments.

## Discussion

### Tissue Phosphorus Response to Temperature Across P Levels

The tissue P concentration observed under sufficient P across the temperature range was comparable to observations made in other studies (Cassman et al., 1981; Walker et al., 1985; Singh et al., 2014). The tissue P concentration of plant and components were largely unaffected by temperature except at the final harvest (i.e., 118 DAP). The distinct temperature response of tissue P concentration between P levels might have led to T × P interactions for plant components at 118 DAP. An increased tissue P concentration under HT observed at 118 DAP was in agreement with a previous report in wheat across P nutrition (Batten et al., 1986; Manoj et al., 2012). In contrast to temperature, P deficiency significantly decreased (≈55% across temperature and plant components) tissue P concentration similarly across harvests and was in agreement with previous observations (Cure et al., 1988; Singh et al., 2014). Under P-deficient conditions, the observed greater allocation of P concentration to leaves than stems appeared to be an adaptation strategy to maximize P utilization by increasing leaf growth (Cure et al., 1988; Singh et al., 2014; Timlin et al., 2017). On average, relatively lower leaf P concentration observed at the final harvest was attributed to the remobilization of nutrients to pods (Hanway and Weber, 1971; Singh et al., 2014).

### Plant Growth, Biomass Partitioning, and Canopy Photosynthesis Response to Temperature Across P Levels

Several growth-related traits and canopy photosynthesis exhibited a pattern of P deficiency-mediated decreases under low and OT, while such decreases were often compensated under warmer temperatures. In addition, the magnitude of temperature response of biomass accumulation and canopy photosynthesis (e.g., C-gain) were often greater under P-deficient conditions. A distinctive temperature response between P treatments was observed for various growth-related traits including, plant height, MSNN, MSLA, TLA, and TDM. For instance, from 22 to 30°C mean daily T (i.e., MLT, OT, and MHT), plant height, and MSNN did not vary substantially under the sufficient P treatment but increased linearly under P-deficient conditions. A curvilinear temperature response of dry matter production under sufficient P nutrition appears to be a common response pattern between the range of temperatures in this study and was also observed in other studies (Wang et al., 1997; Xu et al., 2016). The depressed soybean dry matter and leaf area under P deficiency at or below OT was attributed to the slower developmental rates of plant height (MSER), leaf addition (MNAR), and leaf area expansion (MLAER) and was in agreement with other studies (Fleisher et al., 2012; Singh et al., 2013; Timlin et al., 2017). The decreased height of P-deficient plants was consistent with the decreased MSNN at and below OT, but not under warmer temperatures. Qiu and Israel (1994) also found that P deficiency strongly affects leaf expansion and total canopy area of soybean, which was grown at the OT. However, we found that P deficiency primarily decreased leaf area at and below the OT while warmer temperatures compensated for the decreases.

An independent effect of P deficiency or temperature treatments on biomass partitioning of plant organs has been reported previously for soybean (Cure et al., 1988; Baker et al., 1989; Wang et al., 1997; Singh et al., 2013). However, in this T × P interaction study, we found that the P deficiency appeared to override the temperature response of the biomass partitioning processes, especially at HT. For instance, partitioning of dry matter to plant components tended to be similar at HT between P treatments but differed at lower temperatures. As the temperature increased, the decline of biomass partitioning to pods was in agreement with the study by Wang et al. (1997) and Baker et al. (1989), which reported smaller dry matter partitioning to pods or seeds (harvest index) under warmer temperature in soybean. In fact, pods were not produced at two warmer temperatures (i.e., MHT and HT) at 90 DAP, which might be attributed to the delayed reproductive developments (Thomas et al., 2010). Relative to OT, flower initiation was delayed approximately 8–14 days at warmer temperatures. This might have led to the delayed pod development observed in this study. The temperature effect on days to full bloom (R2 stage) was affected under P deficiency, which took approximately 5–10 extra days relative to the plants grown under sufficient P condition. Delayed flower initiation and pod development in soybean under warmer temperatures have also been reported previously (Baker et al., 1989; Wang et al., 1997). The observed greater biomass partitioning to pods under P deficiency agreed with other studies in soybean grown at OT (Cure et al., 1988).

The canopy carbon exchange rate (CER) measured from the SPAR chambers provides a non-destructive method for determining diurnal photosynthesis, respiration, transpiration, and C-gain throughout the season (Jones et al., 1985; van Iersel and Kang, 2002; Fleisher et al., 2009). Since the majority of plant dry matter (>90%) is derived from photosynthesis, the CER often closely relates with the seasonal dry matter production (Reddy et al., 1991; Fleisher et al., 2008; Timlin et al., 2017). A close association (*r*^2^ = 0.75) between the seasonal total net C-gain and above ground total dry matter production indicated that CER measurements were an excellent indicator of plant growth (**Figure 8**). The slope (0.49) of the linear regression is a measure of the amount of carbon (g) assimilated via canopy photosynthesis that was incorporated into the plant dry matter (g). In other words, the relationship indicated that the carbon fixed from *P*_net_ accounted close to half (49% or 0.49 g C g^-1^ dry matter, DM) of the total above-ground dry matter. Since, whole-plant C concentration of soybean was approximately 40.3% across treatments (data not shown) in this study, the slope of 0.49 suggests that gas exchange values were slightly over-estimated. Nevertheless, this result was comparable to the findings from other researchers using canopy CER measurements in potato (0.40–0.46 g) across CO_2_, water, and temperature regimes (Timlin et al., 2006; Fleisher et al., 2008), soybean (0.50 g) across water stress (Jones et al., 1985), and in the pancy (*Viola × wittrockiana* Gams.) (0.47) across a range of mineral nutrition (van Iersel and Kang, 2002).

The canopy gross photosynthesis (*P*_G_) and evapotranspiration followed a typical diurnal trend of solar radiation (PPFD) (Timlin et al., 2007; Fleisher et al., 2008). During midday or near the saturating or highest PPFD, the *P*_G_ or *P*_G_1500 and evapotranspiration data exhibited the lowest values under the coldest (i.e., MHT) temperature of this study, regardless of P treatments. The largest evapotranspiration values were often observed at warmer temperatures. Evapotranspiration tends to be at its highest under high temperatures but is also influenced by the depth of canopy and total leaf area (Jones et al., 1985). The highest value of *P*_G_1500 varied between the OT and warmer temperatures and showed a curvilinear relationship across P levels. The OT was often accompanied by the greatest growth and leaf area development allowing larger carbon accumulation via photosynthesis due to increased light interception (Reddy et al., 1991). This temperature effect was also observed under sufficient P conditions. However, under P deficiency, MHT appeared to show the greatest value of *P*_G_1500, which was also accompanied with higher growth rates (e.g., MSER, MNAR) and leaf area development.

The weekly net C-gain derived from the canopy net photosynthesis throughout the season showed the highest rate of C-gain around the 12th week (between 80 and 90 DAP) and dropped steeply thereafter (**Figures 6** A,B). Cloud cover and decreased solar radiation (PPFD) around this period appeared to have a major influence on the observed steep drop in C-gain. The first substantial difference in C-gain among temperature treatments was observed the forth week after planting in sufficient P condition, while it began to show within the second week under P-deficient condition and continued almost throughout the season. This observation was also consistent with the significant treatment effects observed for total leaf area and dry matter production 29 DAP. The weekly *R*_D_ followed a similar trend but the decreases at the end of the season was not as steep as C-gain. The greatest *R*_D_ observed at warmer temperatures, especially under P deficiency, reflects negative carbon balance, and is expected due to increased metabolic activities at the HT. Similarly, increased respiration have been found in other crops including soybean under HT (Timlin et al., 2006; Xu et al., 2016) and P deficiency (Singh and Reddy, 2016).

The study illustrated that despite over 50% reduction in tissue P concentration under P deficiency, soybean was able to maintain total net-C-gain and dry matter accumulation close to the control P supply under warmer temperatures (MHT and HT). This compensatory effect indicates that soybean can benefit from larger growth and higher carbon assimilation at warmer temperatures even when plant tissue P concentration is considered deficient (e.g., ≈2.5 versus 5.8 mg g^-1^ of the sufficient P nutrition). In addition, the P utilization efficiency of carbon assimilation (e.g., IPUE_T_*_P_*_net_) across the temperature regimes in this study also showed a large increase under P deficiency with the greatest increase observed at MHT (147%). The greater P utilization efficiency also indicated the plant’s ability to better utilize tissue P concentration under the P-deficient condition and agreed with the previous observations in soybean (Cure et al., 1988; Singh et al., 2014; Singh and Reddy, 2015). Thus, soybean adaptation strategies to moderate P deficiency under warmer conditions appeared to involve faster plant growth rate and leaf area development, and increased C-gain via canopy photosynthesis while also increasing biomass partitioning to leaves and pods and intrinsic P utilization efficiency.

It is noteworthy to mention that the compensatory effects of warmer temperatures on soybean performance under P deficiency were realized under well-watered conditions and a constant replenishment of the root-zone P concentration. In the view of nutrient limitations such as P deficiency on the crop performance, it is important to consider both the root-zone P concentration at a given stage of plant development and the pool of total P that will be available to support plant growth for the entire season (Sinclair, 1992). Under the current experimental conditions, in the P-deficient treatments, the reduced root-zone P concentration was maintained throughout the season via fertigation. Remarkably, the tissue P concentration did not vary substantially among the temperature treatments at a given P level. This was also confirmed by the lack of temperature effect on tissue P concentration across the P treatments for most of the growing season. This suggested a maintained availability of P nutrient in the root-zone. Therefore, when warmer temperature coincides with water stress and/or with lower plant total available P to limit plant growth, a distinct temperature response of soybean growth might occur under P-deficient condition.

## Conclusion

The study revealed for the first time that soybean response to the interactive effects of season-long exposure to temperatures and P nutrition (or levels of tissue P concentration) was altered and largely consistent for the growth-related traits and canopy photosynthesis. For instance, under sufficient P supply, colder or warmer temperatures than optimum tended to decrease canopy net C-gain and dry matter production. However, under a P-deficient condition, relative to OT, the net C-gain and dry matter production decreased only at the moderately low temperature treatment (i.e., MLT). In addition, relative to the OT, the warmer temperatures appeared to increase growth-related traits such as rates of stem elongation, node addition, leaf area expansion, and net C-gain under P deficiency. An increased intrinsic P utilization efficiency of canopy photosynthesis (IPUE_T_*_P_*_net_) across temperature treatments indicated plants can better utilize tissue P concentration under P deficiency. The reproductive growth results showed a different response pattern than observed for dry matter production and C-gain, exhibiting a delay in the anthesis and pod development and a consistent decline of biomass partitioning to pods as temperature increased across P nutrition. While the compensatory effects of warmer temperatures under P deficiency and adaptations shown by soybean, particularly partitioning and P utilization efficiency, are impressive, there remains a significant potential that high temperatures could have severe effects on soybean reproductive development. Overall, the T × P interaction in our experimental condition revealed that the warmer temperature offsets the losses in total dry matter production and net C-gain due to P deficiency. Since P fertilization is a cost-intensive practice in crop production and potential risk of water pollution, the compensatory effect of warmer temperature on the soybean total dry matter accumulation under P deficiency has important agronomic and environmental implications.

## Author Contributions

SS and VR conceived the experiment. SS designed and conducted the experiment, collected and analyzed the data, and wrote the manuscript. DF and DT assisted in SPAR chamber canopy data acquisition and analysis. All authors revised and approved the final manuscript.

## Conflict of Interest Statement

The authors declare that the research was conducted in the absence of any commercial or financial relationships that could be construed as a potential conflict of interest.

## Abbreviations

C-gain: carbon gain
DAP: days after planting
HT: high temperature
IPUE_T_*_P_*_net_: intrinsic P utilization efficiency of total *P*_net_
MHT: moderately high temperature
MLT: moderately low temperature
MLAER: mainstem leaf area expansion rate
MNAR: mainstem node addition rate
MSER: mainstem elongation rate
MSLA: main stem leaf area
MSNN: main stem node number
OT: optimum temperature
P: phosphorus
PAR: photosynthetically active radiation
PPFD: photosynthetic photon flux density
*P*_G_: gross canopy photosynthesis
*P*_net_: net canopy photosynthesis
*P*_G_1500: *P*_G_ at the 1500 μmol m^-2^ s^-1^ PAR
